# Cognitive Impairment-Associated Risk Factors of Parkinson’s Disease: A Hospital-Based Study in a Cohort of Upper Egypt Parkinson’s Patients

**DOI:** 10.3390/brainsci15050459

**Published:** 2025-04-27

**Authors:** Eman M. Khedr, Khaled Aboshaera, Ahmed A. Karim, Mohammad A. Korayem, Gellan K. Ahmed, Doaa M. Mahmoud

**Affiliations:** 1 Department of Neurology and Psychiatry, Faculty of Medicine, Assiut University, Assiut 71511, Egypt; khalednao26@aun.edu.eg (K.A.); mohammadkorayem@aun.edu.eg (M.A.K.); gillankaram@aun.edu.eg (G.K.A.); doaa_mokhtar@med.aun.edu.eg (D.M.M.); 2 Department of Neurology and Psychiatry, Faculty of Medicine, Aswan University, Aswan 81511, Egypt; 3 Department of Psychiatry and Psychotherapy, University of Tübingen, 72070 Tübingen, Germany

**Keywords:** Parkinson’s disease, dementia, risk factors, akinetic rigid, MDS-UPDRS, transcranial magnetic stimulation, resting motor threshold

## Abstract

**Background/Objectives:** Cognitive impairment (CI) in Parkinson’s disease (PD) is a major burden and significantly affects patients’ quality of life. Previous studies found that older age at onset and presence of the akinetic–rigid (AR) subtype are associated with an increased likelihood of CI in PD. The present study aimed to assess factors that are related to the development of CI in PD. **Methods:** Eighty-three PD patients were consecutively recruited. Demographic information, clinical details, Montreal cognitive assessment (MoCA), Movement Disorder Society Unified Parkinson’s Disease Rating Scale (MDS-UPDRS), walking speed, and instrumental activity of daily living (IADL) were assessed. Resting motor threshold (rMT), was also assessed for subgroup of patients with versus without cognitive impairment. **Results:** According to the MoCA cut-off score of 26, 45 had PD without CI (PD-NCI) (54.22%) and 38 cases (45.78%) had PD with CI (PD-CI). The age and age at onset were significantly older in the PD-CI group (*p* = 0.006 and 0.018, respectively). The patients were reclassified into AR and tremor-dominant (TR) phenotype. PD-CI patients were more likely to have the AR (81.6%). Walking speed, MDS-UPDRS score, and IADL scores were significantly worse in PD-CI than in PD-NCI. Stepwise linear regression analysis of risk factors associated CI revealed that higher MDS-UPDRS scores, later age of onset, and higher rMT values were considered risk factors for developing CI. **Conclusions:** Higher UPDRS score, later age of onset, and higher rMT values were considered as risk factors associated CI in PD patients and provide valuable insights for further investigation and potential clinical considerations.

## 1. Introduction

Parkinson’s disease (PD), impacting more than 6 million individuals worldwide, is the second most common neurological illnesses globally. In fact, in the last 30 years, the prevalence of Parkinson’s disease has increased by 2.5 times, making it one of the leading causes of neurological impairment [1]. A crude prevalence rate of PD ranging from 436 to 557 per 100,000 was recorded in Qena and Assiut Nile Valley governorates/Egypt, respectively, which is higher than many other countries [2,3]. Egyptians with PD have a higher prevalence of the Leucine-rich repeat kinase 2 (LRRK2) variant compared to the global average [4]. This may reflect unique environmental, genetic, and healthcare-related factors inherent to the region, where the predominance of agricultural and rural lifestyles, potential exposure to environmental toxins, and variations in healthcare access may contribute to disease onset, progression, and outcomes, including dementia.

In addition to its primary motor characteristics, including bradykinesia, stiffness, and resting tremor, PD is linked to a diverse array of non-motor symptoms that significantly enhance the overall disease burden. CI occurs up to six times more frequently in individuals with PD than in the healthy population [5].

Khedr and colleagues reported that about one-quarter of patients had CI [6,7], while in other studies, it was reported to be 30–40% [8]. There is significant variability in the onset of CI; some people experience its onset within a few years post-diagnosis, while others stay free for decades [5].

Although CI is relatively common in PD and can potentially occur at any disease stage [5], the pathophysiological mechanisms and associated risk factors for developing decline CI in PD remain elusive.

The present study aimed to identify the prevalence of cognitive impairment and clinical and neurophysiological data that might be used to differentiate between PD with cognitive impairment and without, and also to detect risk factors for developing CI in a large cohort of Egyptian patients with PD.

Prior research indicates the early degeneration of dopaminergic neurons in the substantia nigra and the anomalous accumulation of α-synuclein in Lewy bodies in all individuals with PD. In PD patients with concurrent CI, α-synuclein deposition and synaptic pathology are additionally observed in limbic, brainstem, and cortical areas [9,10].

We therefore assume that identifying resting motor threshold (rMT) with transcranial magnetic stimulation (TMS) could potentially differentiate between PD with CI and without.

**Figure 1:**
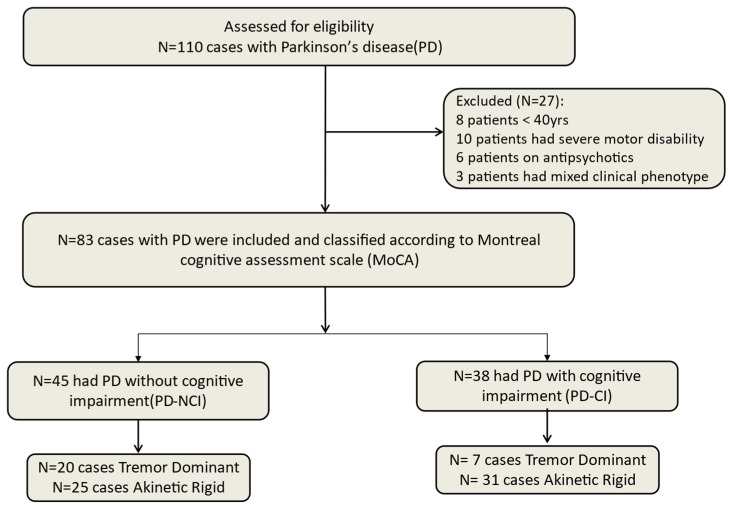
Flowchart of patient recruitment with the inclusion and exclusion criteria of the studied cohort including patient classifications according to the presence of cognitive impairment and the clinical phenotype.

In a recent meta-analysis, hallucinations, older age, and severe motor disability were the most important risk factors associated with the development of CI in PD patients [11]. In this study, we applied correlation and hierarchical logistic regression analyses to identify possible risk factors associated with the development of CI in a large cohort of PD patients in Egypt. Figure 1 illustrates the flowchart of this study.

## 2. Materials and Methods

One hundred and ten patients with PD according to MDS clinical diagnostic criteria for PD [12] were recruited consecutively from the neurology outpatient clinic at Assiut University during the period from May 2023 to May 2024. While Assiut University’s neurology outpatient clinic is a tertiary center, it serves as a centralized hub for medication distribution across the region, receiving a wide range of PD cases. Thus, while selection bias is possible, the clinic’s broad catchment helps mitigate this risk. We also clarified that patient inclusion was strictly based on standardized criteria. Eligibility criteria included a clinical diagnosis of idiopathic PD with gait disturbance, age above 40 years, with stable medication (anti-parkinsonian drugs that have not been changed in at least the last 3 months).

Exclusion criteria were an age below 40 years, presence of other neurological or psychiatric disorders, the use of antipsychotics, a mixed form of clinical phenotype (defined as patients exhibiting clinical features that do not clearly align with either the akinetic–rigid (AR) or tremor-dominant (TD) subtypes of PD according to the Movement Disorder Society), or inability to give informed consent. Patients with significant motor disabilities that may have interfered with this study procedure were also excluded.

Accordingly, 27 patients were excluded: 8 patients aged below 40 years old, 10 patients with severe motor disability (wheelchair), 6 patients on antipsychotics, and 3 patients with a mixed form of clinical phenotype; the remaining 83 participated in this study (see flowchart Figure 1).

### 2.1. Clinical Scales

All patients (N = 83) were assessed with the following clinical scales:

The Montreal cognitive assessment (MoCA) [13] is a widely used tool to screen for CI by assessing attention, concentration, executive functions, memory, language, visuoconstructional skills, conceptual thinking, calculations, and orientation. Nasreddine et al. established that a cut-off score of <26 is a reliable marker for cognitive deficits across various neurological conditions [13]. In the current study, patients were classified into a PD-NCI group (MoCA score ≥ 26) and PD-CI group (MoCA score < 26 including PD-CI either with mild cognitive impairment or more) [13]. The evaluators were blinded to avoid selection bias.

The Movement Disorder Society Unified Parkinson’s Disease Rating Scale (MDS-UPDRS) [14] consists of four sections: the first part assesses the non-motor aspects of everyday living experiences; the second part assesses the motor aspects of daily living experiences; the third part assesses the motor examination; and the fourth part assesses motor complications. This process is used for every patient. To classify patients into the tremor-dominant (TD) and akinetic–rigid (AR) subtypes, we adopted the non-specific tremor criterion as outlined in the Movement Disorder Society Unified Parkinson’s Disease Rating Scale (MDS-UPDRS). Specifically, the classification was based on the relative contributions of tremor and rigidity scores derived from MDS-UPDRS parts II and III. Eleven items for TD and 15 items for AR were selected and developed TD/AR ratio scores for all patients as follow: TD/AR ratio = TD score/AR score; AR score = sum of rigidity items/15; TD score = sum of tremor items/11 [15]. Tremor-related scores were calculated as the sum of all tremor items, while rigidity-related scores were based on rigidity items. The TD/AR ratio was calculated, with cut-off values as follows: TD (≥0.82), AR (≤0.71), and mixed forms (0.71–0.82) [16].

The FOG-Q has been established as a reliable and valid instrument for evaluation of the Freezing of Gait (FOG) in Parkinson’s disease, consisting of six questions and producing a total score ranging from 0 to 24 [17].

The timed up and go test assesses fundamental mobility skills, requiring the patient to stand up from a conventional armchair, move to a predetermined line 3 m away, turn, return, and sit down, with the time taken in seconds used to determine the score. A wristwatch with a second hand was utilized for timing [18].

The 10-Meter Walk Test (10 MWT) is a popular method that was validated across different populations [19] to evaluate gait speed in people with gait limitations. The test consists of 2 m acceleration and deceleration zones, with gait timing performed over the inner 6 m zone. The test consists of 2 m acceleration and deceleration zones, with gait timing performed over the inner 6 m zone. Two paces are assessed: the patient’s average pace, performed three times, and the fastest pace, also performed three times, with averages calculated for each.

The Instrumental Activities of Daily Living [18], a clinical scale devised by Lawton and Brody in 1969, gauges functional independence in tasks beyond basic self-care, like meal preparation and financial management. Widely applied, it is a concise measure useful for assessing independence in various contexts [20].

Measuring the resting motor threshold (rMT), with transcranial magnetic stimulation (TMS):

Due to resource limitations and the logistical challenges of conducting detailed neurophysiological assessments, in a subgroup of N = 61 PD patients, in which 32 had PD-NCI and 29 had PD-CI, the rMT was measured as a crucial parameters of cortical excitability [21]. Patients included in the TMS subgroup were selected based on their ability to comply with the protocol’s requirements, such as remaining relaxed and seated for extended periods, ensuring reliable and reproducible results.

A 3 cm diameter circular ground was placed on the wrist, and silver–silver chloride surface electrodes were used to record electromyography (EMG) signals from the right abductor pollicis brevis (APB) utilizing a muscle belly tendon arrangement. Signals were gathered using a Nihon Kohden Machine type 9400 (Tokyo, Japan). The EMG parameters included a 200 ms recording window and a band pass of 20–1000 Hz. A Magstim 200 magnetic stimulator (Magstim Company Ltd., Sheffield, UK) was attached to a 70 mm figure of eight coil for TMS.

After locating the motor “hotspot” for the left hemisphere’s APB, resting motor thresholds (rMT) were calculated. Prior to stimulation, the EMG signals were tracked and recorded for 20 ms. When the muscle is at rest, the rMT is the lowest TMS intensity required to elicit a predetermined motor-evoked potential (MEP) in the contralateral APB in at least 50% of ten trials [22].

### 2.2. Statistical Analysis

The IBM Statistical Package for Social Sciences, version 26.0 (Armonk, NY, USA: IBM Corp.), was used to analyze the data. The qualitative aspects were described using percentages and ratios. The data distribution’s normality was investigated using the Shapiro–Wilk test. The mean, standard deviation, median, and the Interquartile Range (IQR) were used to describe quantitative data. Spearman correlation between MoCA score and demographic/clinical rating scales, and cortical excitability in all participant cases were assessed. Also, stepwise linear regression analysis was applied to identify risk factors associated with cognitive impairment in PD patients.

## 3. Results

One hundred and ten patients with PD were recruited consecutively, and according to the inclusion and exclusion criteria, only 83 were included in the analysis, as mentioned in the Materials and Methods Section. According to their MoCA score, patients were classified into two groups: PD-NCI group (45 cases, 54.22%) and PD-CI (38 cases, 45.78% of which included mild cognitive impairment and dementia). The age and age at onset in the PD-CI group were significantly higher compared with the PD-NCI group (*p* = 0.006 and 0.018, respectively). Females were significantly more than double in PD-CI compared with PD-NCI. Years of education and number of years of educated patients were significantly lower among PD-CI patients than PD-NCI (*p* = 0.001 for each), while there were no significant differences in the duration of the disease, or family history. According to the MDS-UPDRS scores, 27 patients belonged to the clinical phenotype tremor-dominant (TD), while 56 patients belonged to the AR subtype. There was a lower incidence of the TD type in PD-CI (18.4%) than in PD-NCI (44.4%), and a predominance of the AR type (81.6%) in PD-CI (Table 1A).

Only 61 Parkinson’s patients (32 PD-NCI and 29 PD-CI) were available for assessment of rMT. Analysis of this subgroup were performed for demographic and clinical data with the same significant between PD-NCI and PD-CI group in different parameters as original group indicating no selection bias for the subgroups. rMT showed higher rMT in PD-CI compared with PD-NCI group (*p* value = 0.017), suggesting lower cortical excitability in PD-CI (Table 1B).

Performance on the timed up and go and the average self-selected velocity (m/s) in the 10 m walk were significantly worse in PD-CI than PD-NCI. The scores of parts of the MDS-UPDRS and staging were significantly worse in the PD-CI group. IADL was significantly more affected in PD-CI than in PD-NCI (Table 2).

When TD and AR groups were compared, no clinically significant differences could be found regarding sex, age, years of education, duration of disease, or family history, indicating that none of these factors can account for the high incidence of the AR phenotype in PD-CI (Table 3).

The presence or absence of affected family members had no role in the high incidence of AR phenotype in PD-CI despite finding a small difference in duration of education (*p* = 0.039) (Table 4).

Correlation analyses of all patients (83 cases) revealed that cognitive performance measured by MoCA had negative association with age in years, age of disease onset and AR score. Gait scores, particularly TUG-T, 10 MWT, average self-selected and fast velocity (m/s), total MDS-UPDRS, and AR score were significantly correlated with MoCA score, indicating that motor disability and AR phenotype had greater association with more decline in cognitive function. These findings highlight the importance of considering age, age of onset, motor disability, and motor subtypes (AR) when assessing the risk of cognitive impairment in PD patients. rMT was negatively correlated with MoCA score (*p* = 0.009). A high rMT inversely correlated with MoCA score indicates a low cortical excitability associated with CI (see Table 5).

Stepwise linear regression analysis for risk factors of CI in PD according to MoCA scores were applied to study the effect size of risk factors associated with the development of PD-CI. The final model explained 55.9% of the variance in cognitive performance (MoCA scores). Higher UPDRS scores, later age of onset, and higher rMT values were the strongest risk factors for developing cognitive decline in PD patients (see Table 6).

Intriguingly, rMT had a significant negative correlation with MoCA score in Parkinson’s patients (61 patients) with *r* = −0.334, *p* = 0.009.

The final model explained 55.9% of the variance in cognitive performance (MoCA scores). Higher UPDRS scores, later age of onset, and higher rMT values were the strongest predictors of cognitive decline.

## 4. Discussion

This is the first study on an Egyptian population investigating risk factors associated with CI in PD. Nearly half of the patients recruited into this study had CI. This is likely due to our inclusion of patients with both mild cognitive impairment and dementia under the umbrella of “PD-CI”, which we believe better reflects the clinical spectrum of cognitive decline in PD. This partially explained why our percentage of cognitive impairment is slightly higher than in other studies. In addition, the prevalence of CI in PD varies widely across studies, with some studies reporting that around 30–40% of people with PD develop CI [23].

The higher proportion of females in the PD-CI group (39.5%) compared to the overall PD group (15.6%) emphasizes that sex differences may influence the progression and cognitive profile in PD. Interestingly, although PD is more prevalent among males, females may experience faster cognitive decline. Additionally, a significant portion of female patients in the PD-CI group were illiterate (13 out of 17), suggesting that lower education may be a contributing factor. This underscores the need to consider gender-specific and educational influences in future studies.

Despite a similar disease duration, the PD-CI group were older and had a later age of onset than the PD group (*p* = 0.006 and 0.018, respectively). As the age and education are likely contributors to cognitive outcomes, these factors were significantly different between groups and are acknowledged as potential confounders that should be accounted for in future analyses. They were also predominantly of the AR phenotype (81.6%) and had a worse score in the UPDRS. Inheritance (family history) did not differ significantly. Late onset and older age of patients were considered as risk of developing CI in PD. This outcome was in line with a research that found patients with late-onset PD had a higher risk of CI than those with early-onset PD [24]. Additionally, another study demonstrated that age is a separate risk factor for PD with cognitive impairment [25]. Neuropathological studies might explain why aging is involved in the development of CI as a strong link between age-related accumulation of Lewy bodies in cortical regions and the development of PD-CI was recorded [26]. Additionally, it has been suggested that there is a potential synergistic effect of cortical amyloid-β deposition and aging in the development of PD-CI [10]. Furthermore, several studies have identified the MAPT genotype, involved in tau protein regulation, as a significant genetic susceptibility factor for PD-CI [27,28]. Notably, in Goris et al., PD patients with the H1/H1 genotype among MAPT haplotypes exhibited an age-dependent acceleration in cognitive decline [29]. Collectively, these findings suggest that the interplay of age-related pathological processes, including Lewy body formation, amyloid-β deposition, and genetic predisposition, contributes to the increased vulnerability to PD-CI with advancing age.

An interesting finding in the present study is that patients with PD-CI had a higher incidence of the AR clinical type even though there was no difference in demographic factors or inheritance between AR and TD subtypes. This is consistent with Kann et al.’s groundbreaking research, which showed that reduced grey matter volume and changed functional connectivity are linked to AR symptoms. For the first time, they showed that, unlike tremor symptoms, AR symptoms exhibit unique patterns of anatomical deterioration linked to altered functional connectivity [30]. Furthermore, the pathogenesis of PD has been linked to dopamine depletion’s effects on basal ganglia–cortical interactions as well as a decrease in putamen volume and activation [31]. It has been claimed that AR symptoms are connected with impairments in motor planning dependent on a frontal–parietal network comprising the posterior parietal cortex, pre-supplementary motor region, and premotor cortex, which projects to the motor cortex [32,33]. Wojtala and colleagues also noted the close relation between Parkinson’s motor subtype and cognition [23]. This may be the result of a more widespread reduction in cortical and subcortical surface area, particularly in the left superior parietal gyrus, the left paracentral lobule, and bilateral superior frontal gyrus together with alterations to functional connections in similar regions [34].

This has been supported by several neuroimaging studies associating the AR symptoms with changes in activity, connectivity, and volume of these cortical networks [35,36]. PD patients with greater AR symptoms might be at risk for a more rapid cognitive decline than patients with a TD subtype. Exploring the role of specific genes, particularly race-specific ones and their interactions, in shaping patients’ cognitive reserve or the differences in motor and non-motor symptoms in PD could provide valuable insights and a potential explanation for disparities in disease presentation and progression [37,38,39,40].

Patients with PD-CI performed significantly worse on timed up and go, average self-selected 10 m walk velocity (m/s), and MDS-UPDRS. This reflects the close association between decline in cognitive function and in motor function, particularly rigidity and gait disturbance, similar to findings in a cross-sectional study of Sousa and colleagues [41]. Another study showed that patients with PD-CI more commonly show postural instability and gait disturbance [42]. This might relate better to the fact that the PD-CI patients rather belong to the AR subtype. As expected from the preponderance of the AR subtype, patients in the PD-CI group were more likely to have symptoms of postural instability and gait disturbance. They performed worse on MDS-UPDRS as well as in quality of life.

Stepwise linear regression analysis to assess factors associated CI in PD revealed that higher UPDRS scores, later age of onset, and higher rMT values were the strongest risk factors for developing cognitive decline in PD patients when controlling for age and clinical phenotypes. The findings of present study underline the importance of the hypothesis that both cognitive and motor functions recruit common brain regions and networks that are progressively affected by vascular and neurodegenerative factors [43]. This is assumed to result from the fact that frontal subcortical networks controlling motor and cognitive functions are near to each other, and their watershed vascularization makes them more susceptible to brain micro-vascular disease [43].

rMT is used for motor cortical excitability estimation. Cognitive functions, such as working memory, depend on neuronal excitability in a distributed network of cortical regions [44]. Moreover, there is evidence that corticospinal excitability might be correlated with excitability in other cortical regions related to cognition. We therefore aimed to investigate if rMT are related to measures of cognitive performance in PD patients. The observation that rMT negatively correlated with cognitive performance and considering it as one of associated risk factors for developing CI of PD in the present study confirm the finding of previous studies [45] in healthy subjects, as well as those with PD and Alzheimer’s disease [46,47,48]. This result indicates a direct association between motor cortex excitability and cognitive performance. This could be due to the involvement of projections fibers between the motor cortex and other regions of the brain and also, there could be a network effect, as confirmed by Kamble et al., 2022 [49]. Furthermore, rMT may be considered as a biomarker for developing cognitive decline in PD.

PD-CI was associated with a greater decline in quality of life [18], suggesting that cognitive impairment has a detrimental impact on quality of life in PD patients. This is similar to finding of a study by Leroi and colleagues [50], which found that PD-CI adversely affects quality of life. Lawson et al. [51] also showed that CI had a greater impact on QoL with impaired attention being an important determinant of QoL in PD.

Collectively, these findings highlight the multifactorial nature of risk factors associated with PD-CI and suggest that disruptions in daily functioning may be consequences of PD-CI rather than pre-existing risk factors. Nevertheless, several limitations of the present study should be acknowledged. First, While our data show associations between clinical/demographic factors and cognitive impairment, we acknowledge that causal conclusions cannot be drawn. We recommend longitudinal studies to clarify the temporal sequence of these relationships.

Second, the observed imbalance in sex distribution—specifically, a higher proportion of females in the PD-CI group (39.5%) compared to the overall PD group (15.6%)—raises concerns about potential sex confounding. The use of the non-specific tremor criterion based on the MDS-UPDRS, despite being widely used, does not account for tremor specificity, as the UPDRS considers tremor as part of a broader spectrum of motor symptoms. Further research incorporating more specific and validated tremor criteria may yield additional clarity. The sample size, although modest, was constrained by strict inclusion criteria (e.g., confirmed idiopathic PD, stable medications, minimal comorbidities) to ensure internal validity. This limits broader generalizability and encourage replication in larger, more diverse cohorts.

The absence of assessment of short latency afferent inhibition, which has been linked to cholinergic activity and amyloid deposition, is another point of limitation. We recommend future studies to incorporate SAI as a complementary neurophysiological marker of cognitive status in PD. Larger and more diverse cohorts with comprehensive assessment of cognitive function rather than MoCA and cortical excitability are needed in future studies to validate these findings and assess their applicability across different populations and settings. Additionally, the lack of control for potential confounding variables—such as education, comorbidities, and medication history—further limits the interpretation of our findings.

Continued efforts to better understand the pathophysiology and associated risk factors of PD-CI are essential to identifying effective biomarkers and developing early intervention strategies for at-risk PD patients. Moreover, recent research has increasingly emphasized the role of social cognition and executive functioning—particularly the vulnerability of non-motor symptoms—in PD patients [52]. Additionally, executive function plays a pivotal role in emotion recognition decline, with variations observed across sexes and age groups [53]. Future research should integrate these dimensions to provide a more comprehensive understanding of the non-motor symptoms in PD and their impact on patient quality of life.

## 5. Conclusions

Higher UPDRS scores, later age at onset, and elevated rMT values were identified as significant risk factors for cognitive decline in PD, offering valuable insights for future research and clinical applications.

## Figures and Tables

**Table 1:** (**A**): Demographic data of the studied groups. (**B**): Demographic. (**B**): Subgroup analysis of patients (61 patients) who were available for assessment of resting motor thresholds and clinical data of the studied groups.

**(A)**				
	**PD-NCI** **Mean ± SD** **(*n* = 45)**	**PD-CI** **Mean ± SD** **(*n* = 38)**	**Chi or Z Value**	***p*-Value**
Sex				
Male (61 males with 73.5%)	38 (84.4%)	23 (60.5%)	6.05	0.024
Female (22 females with 26.5%)	7 (15.6%)	15 (39.5%)		
Age	57.16 ± 9.66	62.37 ± 9.17	−2.759	0.006
Median (IQR)	57 (11)	63.5 (10)		
Age of onset (years)	52.38 ± 10.2	57.13 ± 10.57	−2.369	0.018
Median (IQR)	52 (13)	58 (11.25)		
Education				
Yes	40 (88.9%)	21 (55.3%)	11.958	0.001
No (illiterate)	5 (11.1%)	17 (44.7%)		
Duration of education (years)	13.2 ± 5.08	7.53 ± 7.37	−3.37	0.001
Median (IQR)	16 (4)	7 (16)		
Duration of disease (years)	4.78 ± 3.71	5.24 ± 5.04	−0.369	0.712
Median (IQR)	3 (5.5)	4 (4)		
Family history				
Yes	25 (55.6%)	16 (42.1%)	1.491	0.273
No	20 (44.4%)	22 (57.9%)		
Clinical type				
Tremor-dominant	20 (44.4%)	7 (18.4%)	6.357	0.01
Akinetic–rigid	25 (55.6%)	31 (81.6%)		
**(B)**				
**Variable**	**PD-NCI** **(*n* = 32)**	**PD-CI** **(*n* = 29)**	**Chi or Z Value**	***p*-Value**
Sex				
Male	24 (75%)	20 (68.96%)	0.276	0.77
Female	8 (25%)	9 (31.04%)		
Age	54.75 ± 8.99	63.21 ± 9.65	−3.34	0.001
Median (IQR)	55 (16)	63 (13)		
Age of onset	49.64 ± 10.00	58.38 ± 10.50	−3.063	0.002
Median (IQR)	51.25 (17)	57 (12)		
Education				
Yes	27 (84.38%)	12 (41.38%)	12.19	0.001
No	5 (15.62%)	17 (58.62%)		
Years of education	12.31 ± 5.37	5.38 ± 6.93	−3.53	0.0001
Median (IQR)	15 (4)	0 (12)		
Duration of illness	5.11 ± 3.83	4.59 ± 4.86	−0.859	0.391
Median (IQR)	4 (6)	3 (3)		
Family history				
Yes	14 (60.87%)	10 (34.48%)	0.547	0.60
No	18 (39.13%)	19 (65.52%)		
Total UPDRS	73.66 ± 36.54	108.28 ± 38.03	−3.438	0.001
Median (IQR)	69 (46)	107 (43)		
Clinical type				
Tremor-dominant	15 (46.87%)	5 (17.24%)	6.06	0.016
Akinetic–rigid	17 (53.13%)	24 (83.76%)		
Resting motor threshold (RMTS)	37.38 ± 7.71	42.55 ± 8.60	−2.379	0.017
Median (IQR)	36 (13)	43 (13)		

**Table 2:** Differences in clinical rating scales between the PD-NCI and PD-NCI groups.

Variables	PD-NCI Mean ± SD (*n* = 45)	PD-CI Mean ± SD (*n* = 38)	Chi or Z Value	*p*-Value
Freezing of Gait Questionnaire (FOG-Q)	2.8 ± 5.91	3.95 ± 5.99	−1.23	0.219
Timed up and go (TUG-T) (in seconds)	48.69 ± 41.02	87.71 ± 62.91	−3.305	0.001
Median (IQR)	32 (53)	71 (84.25)		
The 10-Meter Walk Test (10MWT)				
Average self-selected velocity (m/s)	0.836 ± 0.578	0.485 ± 0.441	−3.099	0.002
Median (IQR)	0.67 (0.64)	0.31 (0.51)		
Average fast velocity (m/s)	1.27 ± 0.764	0.735 ± 0.648	−3.346	0.001
Median (IQR)	1.27 (1.22)	0.58 (0.75)		
MDS-UPDRS Part I: Non-Motor Aspects of Experiences of Daily Living (n-MEDL)	11.756 ± 6.889	19.263 ± 8.15	−4.267	<0.001
Median (IQR)	11 (7)	18 (9.50)		
MDS-UPDRS Part II: Motor Aspects of Experiences of Daily Living	19.711 ± 9.603	28.84 ± 11.103	−3.599	<0.001
Median (IQR)	19 (16.5)	30 (16.25)		
MDS-UPDRS Part III: Motor Examination	50 ± 22.427	66.026 ± 24.18	−2.880	0.004
Median (IQR)	47 (31.5)	60.5 (36)		
HOEHN and YAHR staging	1.93 ± 0.963	2.5 ± 1.225	−2.363	0.018
Median (IQR)	2 (2)	2 (1)		
Instrumental Activity of Daily Living [18]	6.22 ± 1.704	4 ± 1.845	−4.908	<0.001
Median (IQR)	6 (3)	4 (2)		

**Table 3:** Demographic data of the tremor-dominant and akinetic–rigid group.

	Tremor-Dominant Mean ± SD (*n* = 27)	Akinetic–Rigid Mean ± SD (*n* = 56)	Chi or z Value	*p*-Value
Sex				
Male	19 (70.4%)	42 (75%)	0.200	0.654
Female	8 (29.6%)	14 (25%)		
Age	59.00 ± 9.94	59.8 ± 9.72	−0.749	0.454
Median (IQR)	58 (13)	60.5 (9)		
Age group				
Younger than 50 yrs	2 (7.4%)	9 (16.1%)	1.189	0.275
Older or equal to 50	25 (92.6%)	47 (83.9%)		
Age of onset	54.63 ± 10.34	54.52 ± 10.79	−0.224	0.823
Median (IQR)	54 (15)	55.5 (12.5)		
Education				
Yes	22 (81.5%)	39 (69.6%)	1.311	0.252
No	5 (18.5%)	17 (30.4%)		
Duration of education	12.26 ± 6.25	9.8 ± 6.99	−1.702	0.089
Median (IQR)	16 (4)	12 (16)		
Duration of disease	4.37 ± 3.78	5.29 ± 4.6	−1.195	0.232
Median (IQR)	3 (6)	4 (4.5)		
Family history				
Yes	14 (51.9%)	27 (48.2%)	0.096	0.756
No	13 (48.1%)	29 (51.8%)		
PD-NCI vs. PD-CI				
PD-NCI	20 (74.1%)	25 (44.6%)	6.357	0.018
PD-CI	7 (25.9%)	31 (55.4%)		

PD-NCI: Parkinson’s disease without cognitive impairment; PD-CI: Parkinson’s disease with cognitive impairment.

**Table 4:** Demographic data of cases with positive and negative family history group.

Variable	Positive Family History Mean ± SD (*n* = 41)	Negative Family History Mean ± SD (*n* = 42)	Chi or z Value	*p*-Value
Sex				
Male	29 (70.7%)	32 (76.2%)	0.317	0.573
Female	12 (29.3%)	10 (23.8%)		
Age	59.44 ± 10.57	59.64 ± 8.99	−0.160	0.873
Median (IQR)	59 (11)	50.5 (10)		
Age group				
Younger than 50 yrs	6 (14.6%)	5 (11.9%)	1.34	0.714
Older or equal to 50	35 (85.4%)	37 (88.1%)		
Age of onset	53.68 ± 11.4	55.4 ± 9.77	−0.902	0.37
Median (IQR)	53 (16)	55 (11.75)		
Education				
Yes	34 (82.9%)	27 (64.3%)	3.701	0.054
No	7 (17.1%)	15 (35.7%)		
Duration of education	12.05 ± 6.07	9.19 ± 7.27	−2.062	0.039
Median (IQR)	16 (6)	12 (16)		
Duration of disease	5.76 ± 5.37	4.24 ± 2.92	−1.116	0.265
Median (IQR)	4 (5)	3 (4)		
PD-NCI vs. PD-CI				
PD-NCI	25 (61%)	20 (47.6%)	1.491	0.273
PD-CI	16 (39%)	22 (52.4%)		
Clinical type				
Tremor-dominant	14 (34.1%)	13 (31%)	0.096	0.756
Akinetic–rigid	27 (65.9%)	29 (69%)		

**Table 5:** Correlation between MoCA score and demographic/clinical rating scales, and cortical excitability in all participant cases.

	MoCA Score	
**Demographic Data**		
Gender	r	−0.202
	*p* value	0.067
Age in years	r	−0.381
	*p* value	<0.0001
Age of onset	r	−0.323
	*p* value	0.003
Years of education	r	0.428
	*p* value	0.001
Family history	r	−0.096
	*p* value	0.386
Duration of disease (in years)	r	−0.044
	*p* value	0.690
**Gait Scales**		
Freezing of Gait Questionnaire (FOG-Q)	r	0.279
	*p* value	0.089
Timed up and go (TUG-T) (in seconds)	r	−0.357
	*p* value	0.001
The 10-Meter Walk Test (10MWT) Average self-selected velocity (m/s)	r	0.384
	*p* value	<0.0001
Average fast velocity (m/s)	r	0.406
	*p* value	<0.0001
**MDS-UPDRS, IADL, and Clinical Phenotype**		
Total MDS-UPDRS	r	−0.429
	*p* value	<0.0001
Tremor Score	r	−0.151
	*p* value	0.173
Akinetic–rigid Score	r	−0.432
	*p* value	<0.0001
**Cortical Excitability for 61 Patients**		
Resting motor threshold (rMT)	r	−0.334
	*p* value	0.009

**Table 6:** Stepwise linear regression analysis of risk factors related for developing cognitive impairment in Parkinson’s disease according to MoCA scores.

Predictors	Model 1 Nag R^2^ = 0.287		
	*p*-Value	Unstandardized Coefficients (B)	95.0% Confidence Interval for B
Total MDS-UPDRS	<0.0001	−0.094	−0.139–0.048
	**Model 2** **Nag R^2^ = 0.505**		
Total MDS-UPDRS	<0.0001	−0.101	−0.139–0.062
Age of onset	<0.0001	−0.324	−0.477–0.170
	**Model 3** **Nag R^2^ = 0.559**		
Total MDS-UPDRS	<0.0001	−0.089	−0.128–−0.050
Age of onset	<0.0001	−0.280	−0.432–−0.127
Resting motor threshold (stimulation in percentage)	0.033	−0.232	−0.445–−0.020

Total MDS-UPDRS—Movement Disorder Society Unified Parkinson’s disease Rating Scale.

## Data Availability

The original contributions presented in this study are included in the article. Further inquiries can be directed to the corresponding author.
